# The role of communication, building relationships, and adaptability in non-profit organisational capacity for health promotion

**DOI:** 10.1093/heapro/daac074

**Published:** 2022-07-28

**Authors:** Louise A van Herwerden, Dianne P Reidlinger, Claire Palermo

**Affiliations:** Faculty of Health Sciences and Medicine, Bond University, Gold Coast, Australia; Faculty of Health Sciences and Medicine, Bond University, Gold Coast, Australia; Department of Nutrition, Dietetics and Food, Faculty of Medicine, Nursing and Health Sciences, Monash University, Melbourne, Australia

**Keywords:** capacity building, adaptive capacity, health promotion capacity, longitudinal study, non-profit organisation

## Abstract

While the non-profit sector has an integral role in health promotion, it is unclear whether these organisations have the capacity for health promotion activities. This study aims to explore and describe capacity changes of a non-profit organisation during a 3-year community-based nutrition intervention. The non-profit organisation, with 3800 members throughout the state of Queensland, Australia, implemented a 3-year food literacy community-based intervention. A team of qualified nutritionists delivered the program in partnership with community-based volunteers. A separate aim of the intervention was to build capacity of the non-profit organisation for health promotion. A qualitative study was undertaken, using a social constructivist approach to explore organisational capacity changes longitudinally. All relevant participants including non-profit executive managers and nutritionists were included in the study (100% response rate). Data collection included semi-structured interviews (*n* = 17) at multiple intervention time points and document analysis of program newsletters (*n* = 21). Interview transcripts and documents were analysed separately using thematic and content analysis. Codes and categories between the two data sources were then compared and contrasted to build themes. Organisational capacity was predominantly influenced by four themes; ‘communicating’, ‘changing relationships’, ‘limited organisational learning’ and ‘adaptability and resistance to change’. Developing non-profit organisational health promotion capacity appears to require focusing on fostering communication processes and building positive relationships over time. Capacity changes of the non-profit organisation were not linear, fluctuating across various levels over time. Assessing non-profit organisational capacity to implement community interventions by describing adaptive capacity, may help researchers focus on the processes that influence capacity development.

## INTRODUCTION

The non-profit sector is the sum of private, voluntary, non-profit organisations and associations that have become a major economic and social force integral to health provision (Anheier, 2014). Non-profit organisations have been identified as important vehicles and partners for health promotion interventions to create sustainable healthy communities (Smith *et al.*, 2006). Non-profit organisations require capacity to support effective health promotion practice. The term capacity, can refer to organisational, community or individual ability to take effective action (Hawe *et al.*, 1998). Research to date suggests two levels of non-profit capacity; individual expertise that includes the skills, knowledge, and experience that volunteers and staff bring to the organisation and organisational resources and procedures that enable agencies to use individual expertise productively (Schuh and Leviton, 2006; Lawrenz *et al.*, 2018). Organisational capacity is the set of structures and functions a non-profit organisation needs to effectively serve the community (Despard, 2017).

Health promotion capacity building is described as “the development of knowledge, skills, commitment, structures, systems, and leadership to enable effective health promotion” (Smith *et al.*, 2006). Organisational capacity building is promoted as a way to enhance the effectiveness and sustainability of nonprofits, yet there are few published studies on the effects of capacity building on non-profit organisations (Sobeck and Agius, 2007). When non-profit organisations aim to build capacity, the focus is frequently on individual training, to enhance workforce development. Yet, the organisation may not be able to use the increased expertise without some changes in its own processes and resources (Schuh and Leviton, 2006). Researchers have identified potential outcomes related to capacity building, ranging from improving management competencies or diversifying funding to serving more clients or improving sustainability (Connolly and Lukas, 2002; Sowa *et al.*, 2004; DeCorby-Watson *et al.*, 2018).

There are specific theories, models and frameworks identified to support capacity building interventions relevant to public health organisations (Bergeron *et al.*, 2017; van Herwerden *et al.*, 2019). However, few organisational capacity frameworks specifically assess nonprofits levels of capacity (Minzner *et al.*, 2014; Company, 2001) and of those that do, it appears none are validated (Despard, 2017). So although capacity is defined in the non-profit literature, no standardised measures exist, making it difficult to accurately assess organisational capacity (Despard, 2017). One useful framework used to assess capacity changes over time in institutions is the Adaptive Capacity Framework or ‘wheel’. This framework describes organisational adaptive capacity as having six dimensions: variety, learning capacity, room for autonomous change, leadership, availability of resources and fair governance (Gupta *et al.*, 2010). Adaptive organisations encourage actors to learn and allow individuals to question roles, rules and procedures that are important for problem solving.

Adaptive capacity, or adaptability meaning the ability of a system to prepare for stresses and changes in advance or adjust and respond to the effects caused by the stresses (Engle, 2011), may be a useful indicator for non-profit organisational capacity assessment. This concept has been highlighted as an important element for organisational change sustainability (Greenwood-Lee *et al.*, 2016). Organisations are fluid and dynamic and they can sometimes develop or devolve rapidly. In social systems, organisations and networks that learn and store knowledge and experience, create flexibility in problem solving and balance power among interest groups, play an important role in adaptive capacity (Berkes *et al.*, 2008). Adaptive capacities are the social and technical skills and strategies, of individuals and groups, that are directed towards responding to environmental and socioeconomic changes. Adaptive capacity is effectively a feedback loop capturing the response of the system to changes in the environment (factors it cannot control) in which it operates. Hence, measuring adaptive capacity may be an important concept assessing capacity changes in an organisation.

A recent systematic literature review assessing capacity highlighted that further research should explore the importance of adaptive capacity or capacity building processes in community-based public health interventions (van Herwerden *et al.*, 2019). Capacity indicators need to move away from being rigid, inflexible and linear. Practitioners and researchers should embrace an iterative adaptive process, engaging with a wider range of stakeholders, with participatory input and reflective practice processes that enable learning. However, research on how non-profit organisations’ adaptive capacity can be assessed over time is limited. This study aims to explore and describe capacity changes of a non-profit organisation over the course of a 3-year health promotion community-based intervention.

## METHODS

### Setting

The community intervention in this study was implemented by a non-profit organisation, with around 3800 members throughout the state of Queensland, Australia, from 2015 to 2018. Funding was provided by the State government health department to address rising obesity concerns. The multi-strategy intervention aimed to improve the nutritional health of individuals living in regional, rural, and remote Queensland, tapping into over 240 committees and their volunteer members located throughout the state. There were two inter-related but distinct strategic approaches: 1) capacity building of the non-profit organisation for health promotion, with elements of community training, empowerment and support and 2) delivering a food literacy intervention with face-to-face nutrition education and cooking sessions reported elsewhere (Palermo *et al.*, 2018) (Table S1).

A team of qualified nutritionists delivered the program throughout Queensland. The non-profit organisation managed the budget, strategic planning, and governance of the intervention in conjunction with the project coordinator. The program implementation was supported locally by non-profit volunteers. This study is focused on the non-profit organisational capacity changes over time throughout the implementation of the intervention.

### Study design

The researchers used a constructionist approach to explore capacity changes over time, learning from each interview to inform data collection in follow up interviews. The methodology drew on qualitative description (Patton, 2015) to explore and describe capacity changes. Methods were chosen to emphasize the local context and to facilitate reflection with participants involved in the process (Israel *et al.*, 1995; Braun *et al.*, 2014). As such, data collection included qualitative semi-structured interviews with project and non-profit executive management at the start, during and end intervention time points. Learnings from each interview informed data collection for subsequent interviews. Document analysis of newsletters called the *Monthly Munch,* was also conducted to confirm or refute findings from the interviews. The newsletter was published by the organisation monthly over the course of the intervention, describing the evolution of the program and implementation process in the various communities and therefore was chosen as the most appropriate document to describe changes in the intervention and capacity over time. Ethics approval was obtained from the Monash University Human Research Ethics Committee (approval number 7075).

### Sample selection

A complete sampling technique (Patton, 2015) was applied to recruit participants for the interviews. Given the focus on organisational capacity the sample included the 1) nutritionist project manager and nutritionists, who oversaw implementation of the various program strategies, and 2) executive management that were already employed by the non-profit organisation and who managed the budget and organisational operational processes for the program. Volunteers’ views are captured in a separate study. All participants (*n* = 11) were invited by email, with two reminders, to participate in a face-to-face or telephone interview with the researcher at commencement, during and end of the program points. All nutritionists and executive management agreed to participate (100% response rate). Staff turnover during the study was captured by conducting entry and exit interviews with each employee starting (*n* = 3) or leaving the program (*n* = 2) (45% nutritionists’ turnover during intervention). Documents sampled were all [blinded for peer review] newsletters distributed monthly in 2017 and 2018.

### Data collection

A summary of the study data selection is outlined in Table 1. Interviews were the primary data source, with documents (newsletters) a secondary data source.

**Table 1:** Non-profit organisation study data collection, sample size and timeline

Data collection source	2016	2017	2018	Total
Interviews				19
Nutritionists working in case communities (*n* = 8)	3		4	7
Entry nutritionist’s (staff turnover)		3		3
Exit nutritionist’s (staff turnover)	2	1	1	4
Executive management (*n* = 3)	2	2	1	5
Documents				*21*
Newsletters (*n* = 21)		11	10	21

*n* = sample size.

### Interviews

Interviews were conducted within the first year of the intervention (November 2016), mid-way through the intervention (2017) and at the end intervention (May 2018). Entry and exit interviews were also conducted as new staff started or left the program. An interview schedule with open-ended questions was developed and adapted for end intervention interviews (Table S2) to give participants the opportunity to describe their experiences on organisational capacity building elements. The questions were derived from a review of the literature on capacity (MacLellan-Wright *et al.*, 2007; Kostadinov *et al.*, 2015; van Herwerden *et al.*, 2019) staff experiences of implementing an intervention and an organisational adaptive capacity framework (Gupta *et al.*, 2010). Interviews lasted between 24 and 90 min, were audio recorded and transcribed verbatim for later analysis.

### Monthly Munch newsletter documents

All editions of the *Monthly Munch* newsletter (*n* = 21) released every month online on the non-profit organisation website and mailed out to a mailing list, was collated from February 2017–December 2018. The newsletter was compiled by the project coordinator. *Monthly Munch* newsletters consisted of 1) project updates, which included new staff, project reach and travel schedules 2) volunteer facilitators in the spotlight and 3) nutrition education and recipes.

### Data analysis

Interviews were conducted across non-profit executives and all nutritionists, to avoid reliance on single viewpoints or time points. Data were collected and analysed for each year (2016–2018) in separate spreadsheets. Interview analysis descriptions were labelled “nutritionist” or “executive management” for each interview with the year of interview e.g.: nutritionist (2018). Nutritionist and executive management transcripts were labelled numerically to ensure anonymity.

Data was explored using thematic analysis (Braun *et al.*, 2014). Initially, data was inductively coded. Inductive coding was done in Microsoft Excel after which codes were pasted into Nvivo software (version 11, QSR international) as a coding framework for the in-depth analysis (Table S3). Inductive coding of the first transcript was completed independently by all authors to establish the coding framework. After discussions with all authors following coding of the first transcript, coding continued through the remaining interviews using the coding framework. Additional codes were added inductively where identified. To inform early analysis into themes, content analysis was employed whereby the number of codes were counted as frequencies. Analysis was furthered by deductively allocating these codes to categories aligned with a framework developed from a range of capacity literature, which included established capacity domains (van Herwerden *et al.*) and the Adaptive Capacity Framework (Table S3) which were then developed into themes. Thematic analysis involved going backwards and forwards between the years in order to capture capacity changes over time. The primary researchers’ own subjective experiences were logged and reflective dialogue with one other author was conducted during thematic development.

### Monthly Munch newsletter documents

Document analysis (Bowen, 2009) of all twenty-one *Monthly Munch* newsletters was conducted using the previously described inductive coding. The coding process was performed by one researcher, with the assistance of Nvivo software (version 11, QSR international). The researcher went through each newsletter line by line, whereby codes were assigned to pieces of text within the documents. A sample of newsletters (*n* = 2) were coded independently by another researcher to verify the approach. The frequency of codes was counted. Codes from the documents were further grouped into categories (Table S2) which were analysed to identify relationships between categories and to support the development of themes. Themes that described the common non-profit organisational capacity changes that occurred over time were developed from the codes and categories from the documents. The themes from the document analysis and interview transcript analysis were compared and contrasted to support final themes and quotes from interviews, and elements of text from documents were selected to support illustration of findings.

## RESULTS

### Sample size, demographics, and volunteer characteristics

A total of seventeen interviews were included in the analysis. From the total interviews, three staff completed initial and end intervention interviews (*n* = 3). Three new staff or executives completed entry and exit interviews (*n* = 3) throughout the intervention and five staff or executives completed exit interviews only (*n* = 5). This sample represented every individual who was involved in the intervention from its inception to completion. The nutritionists were all females and ranged in age from 23 to 30 years, while the nutritionist project manager was a in her early fifties. The organisation executive management were all females and ranged in age from mid-fifties to late seventies.

### Non-profit organisation capacity changes over time

The most frequently coded deductive categories from the interviews over time were quality project management (*n* = 282 codes), organisational development (*n* = 260) and workforce development (*n* = 144). Several inductive categories were also developed. The most frequently reported of these were travel (*n* = 78) and facilitator characteristics (*n* = 56). The majority of newsletters described elements of food literacy (*n* = 97) (nutrition education, monthly recipes, healthy catering guidelines) and public relations and marketing (*n* = 43). Capacity elements described in the document analysis most frequently over time were community activities (*n* = 79), travel (*n* = 47), partnerships (*n* = 40), workforce development (*n* = 38), quality project management (*n* = 33) and communication (*n* = 26). The capacity categories were analysed over time (2016–2018) and developed into themes and sub-themes (Table 2).

**Table 2:** Key themes describing capacity development of a non-profit organisation to implement community nutrition interventions over time (*n* = 17 interviews)

Theme definition and description	Categories
1. Changing relationships between executive management, nutritionists, and volunteers	Partnerships
	Communication
This theme reflects those changing relationships between executive management, nutritionists and community volunteers were pivotal to capacity development of the non-profit organisation	Quality Project Management
	Community Activities
2. Communication processes during implementing the nutrition program	Facilitator characteristics
This theme reflects the non-profit organisational processes of sharing and sorting of information for the specific purpose of planning and implementing the health promotion community-based interventions	Travel
3. Limiting room for autonomous change in the non-profit organisation	Organisational development
This theme reflects the limited non-profit organisations’ capacity for flexibility and ability to adapt governance structures, policies, operational procedures, and the responsiveness to the people involved (their inputs and costs required) to implement the health promotion community-based interventions	Quality Project Management
	Workforce development
4. Learning organisation culture	Fair governance
This theme reflects that the culture created by the non-profit organisation influenced the learning of the people who worked (executive management and nutritionists) and volunteered there	Learning capacity
	Variety
	Room for autonomous change

The capacity changes described over time were not linear and fluctuated depending on the context described by participants. Capacity changes of the non-profit organization were described as interacting across various levels over time, with several parallel capacity change stories emerging. Capacity changes with the non-profit executive management team and their interactions with the project team, and interactions between the organisation community volunteers with the project team were described.

The factors influencing non-profit organisational capacity to implement health promotion community-based interventions were found to be predominantly influenced by two major themes: relationships and communication. Two sub-themes that also influenced capacity development over time were ‘*limited room for autonomous change*’ in the non-profit organisation and ‘*the learning organisational culture*’. Capacity was found to be either enhanced or limited by change, adaptability, and organisational learning. Relationships and communication processes between executive management, nutritionists and volunteers impacted on the learning organisational culture. These individual and organisational level factors influenced capacity development of the health promotion community-based interventions.

At the individual level, capacity development was influenced by the relationships between the non-profit executive management team and nutritionists. Relationships between the organisational volunteers with the nutritionists also influenced capacity development. At the organisational level, executive management influenced governance structures, policies, operational procedures, and the responsiveness of the people involved in the health promotion community-based interventions. All individuals involved with the non-profit organisation influenced the learning organisational culture. Each of these themes and sub-themes will now be described in detail.

### Changing relationships between executive management, nutritionists, and volunteers

All executive managers and nutritionists described the importance of building relationships with volunteers to successfully implement the community interventions. Initially management described volunteer members being sceptical of the young nutritionists and the intervention, explaining that there was little trust or enthusiasm for the intervention. The nutritionists also described that initially volunteers were dubious, and they felt judged by their age, being too young and hence too inexperienced to run food literacy health promotion programs.

‘We did sort of experience a few personality clashes initially and that was really challenging in house, just to try and get that rapport back within the team’.” ^executive management (2016)^

‘Then you’ve got those complicated dietitians (nutritionists).’ ^executive management (2016)^

Over time, relationships were established as nutritionists visited and ran food literacy programs in many communities in conjunction with volunteers. The [removed for blind review] newsletters provided descriptions of relationships and social connections developing between volunteers and nutritionists over time. Executive management described hearing positive stories from their volunteers about the program staff and this in turn shifted their scepticism as the nutritionists managed to build strong connections with the volunteers. By the end of the intervention, management described the importance of getting young women to join their organisation and that the nutritionists being young and vibrant were what the organisation needs.

“They’ve built the rapport quite quickly because they’re approachable, they’ve just got great personalities, and they’ve realised that we have got an older generation of people out there” ^executive ­management (2018)^

The nutritionists described a strong level of trust that had developed over time with community volunteers. They described empowering and supporting volunteers to implement a food literacy program and continue implementing new local community interventions. It was clear from many nutritionists’ descriptions that they persisted in their role due to the strong relationships they had developed and the encouragement they had from community volunteers. Document analysis also highlighted developing relationships.

“But they’ve had to build that initial rapport. I always say rapport because if you don’t have that, you don’t have trust, well then you’re never going to get anywhere, especially in the non-profit.” ^executive management (2016)^

Relationships between executive management and nutritionists were described as tense and fractious at times, with miss communication, skills and roles not clearly defined and different expectations, creating a tense work environment.

“I’m doing as best as I can, and I don’t do it very well, they need to feel supported, that they’ve got everything that they need to do the job out there, and sometimes that doesn’t work because I get shot or I get maimed from the top and I’m angry and wounded, and then it hits them”. ^nutritionist (2017)^

Several nutritionists described the culture and tension in head office of the non-profit organisation as effecting relationships and contributing to staff turnover. Other staff described that they only stayed in their job because they were frequently out on the road and hence relieved not to be in the office. Executive management themselves acknowledged the non-profit organisation as extremely complicated, highlighting the different personality types which made implementing a community intervention very difficult.

Relationships between executive management and community volunteers were also described as fractious. Nutritionists described the lack of organisational support at the community level, primarily due to a lack of organisational supports with perceived rigidity of policies and procedures, which were not flexible to accommodate the implementation of community nutrition interventions.

‘I think we’ve got so many excellent facilitators that show awesome leadership and have been resourceful and really excelled, but without that organisational support it’s almost like you see their wings being clipped a little bit and it makes you worry for the future of their ability.^’ nutritionist (2018)^

‘Obviously, the success of this program has been quite dependent on the relationship between the facilitator and the branch president. Where you’ve got a branch president engaged, then the facilitator is more likely to feel supported and to run the program.’ ^executive management (2016)^

### Communication processes during implementation of the nutrition program

This theme reflects the non-profit organisational processes of sharing and sorting of information for the specific purpose of planning and implementing the health promotion community-based interventions. Communication styles included the imparting and exchange of information by verbal, nonverbal, written, and visual means, which impacted on the working relationships between the executive management, nutritionists, and community volunteers. Communication and approval systems within existing organisational governance structures were a hindrance to capacity development within the health promotion community-based interventions.

All non-profit management and nutritionists described various issues with communication, with more frequent issues described over time. These included structural barriers to communication, poor quality management communication processes, nutritionists and non-profit differing organisational styles (health industry versus non-profit communication style) as well as individual differences in communication styles. Miscommunication resulting in fractious relationships between nutritionists and the executive management of the non- profit organisation were described as deteriorating over time by all participants.

“It had more of an effect on us as a project team as well…. having to fight that battle all the time of trying to get support and trying to get good communication and good understanding it has worn our spirit down a little which is sad.” ^nutritionist (2071)^

^“^I think, like communication with the organisation has always been our biggest challenge. Things, miscommunication or incorrect communication has been a barrier or roadblock along the way.’ ^nutritionist (2018)^

At the start of the health promotion food literacy program, quality project management communication issues were most frequently described. Nutritionists described not feeling heard or supported when challenges were raised with management. Staff also described not being involved in communication processes, such as management meetings, where there were program implementation decisions made that impacted on them.

“I think it was a strategy to not have us in there because this communication was so poor and nothing was getting done^.” nutritionist (2016)^

“Not just the logistical breakdown of physical channels of communication, but the language, and people misinterpreting” ^nutritionist (2016)^

The travel schedule was another area highlighted by program staff as creating communication issues. They explained that being constantly out on the road, so not in the office, resulted in hearing information second hand or missing out on information all together, due to it not being sent via email or documented in a transparent way. Travel was also frequently mentioned in the [removed for blind review] newsletters with a heavy emphasis on reporting where staff were travelling, particularly in 2017 newsletters.

The communication issues at the 2018 interviews were most frequently described as due to structural issues resulting in miscommunication with confusion about how, when and to whom to communicate with around project management specifics. This was influenced by large staff turnover throughout the intervention across all levels of management and nutritionists, with over 90% staff turnover rates described.

“But I think sometimes the program management of the program affected the team a lot. Even the state manager and the culture within state office really affected the team a lot. I think that’s why people left.” ^nutritionist (2017)^

“I came in July 2016, and I was the fifth team member they’d employed at that point, because travel was getting so hectic. But I think sometimes the program management of the program affected the team a lot. Even the state manager and the culture within state office really affected the team a lot. I think that’s why people left.” ^nutritionist (2018)^

Relationships with community volunteers and state office staff influenced communication. Those community volunteers who had been with the non-profit for many years were described as having a better understanding of the communication processes and were able to reduce miscommunication. Volunteers who had more effective communication strategies, such as being direct, going around the red tape and speeding up what were described as very slow bureaucratic organisational communication processes had better communication strategies.

Several volunteer facilitators did appear to communicate about the program with their organisation by preparing branch reports, attending organisation division meetings and conferences highlighting the intervention (as described in document analysis). The nutritionists also set up a [removed for blind review] Facebook Group as a communication tool for volunteer facilitators to connect remotely and support each other, share resources and community activities.

“Thanks to all the branches who sent in their branch reports for the month of September.” ^Newsletter October 2018^

Overall, the non-profit organisation’s verbal and written communication channels descriptions had a negative impact on capacity development for the health promotion food literacy program over time. There were several descriptions of a shift with some improvements in verbal communication between executive management and nutritionists by mid 2018. However, this was at the end of the funding phase for the health promotion food literacy program.

“I’m just grateful that the message is getting out through our division presidents as well, because a lot of them were very sceptical. But from this board meeting we’ve just had, I would say all of them, all 18 are on side now for Country Kitchens, and really want to work for the future.” ^executive (2018)^

I think it’d be at least another 3 years until there was significant organisational support and understanding with better communication having a long way to go.’ ^nutritionist (2018)^

### Limiting room for autonomous change in the non-profit organisation

There were reports of too much change regarding staff turnover, policy and procedural changes, program implementation changes and travel schedule changes. Too much change was perceived to create instability and on occasions chaos. Staff described that the high staff turnover impacted on the capacity of the non-profit to implement the community intervention.

“This year marked big changes for the food literacy program.” ^Newsletter December 2018^

Yet the organisation, from executive management to community volunteers, were frequently described as being resistant to change.

“Just trying to change recipes that have been around for hundreds of years, too hard.” ^nutritionist (2018)^

The non-profit organisational structure was often described as inflexible, limiting autonomous change along with limited vision and leadership for the organisation to move towards implementing strategic policies and procedures to support all members to act as health promotion agents. Although there were some accounts of visionary people, the structure of the organisation which dictated a change of president and other roles every two to 3 years meant that even if there were strong leaders, people were only able to lead for a limited time. Data indicated the intervention was not strategically supported, with staff stating no intervention strategies were translated into policies and strategic practices.

Nutritionists and volunteers were described as adaptable. The staff developed a food literacy program that they piloted and adapted for different communities over the intervention period. There were also descriptions about the flexibility of nutritionists with regard to project management operational procedures such as changes in travel plans, leave request forms and reporting structures. Staff also described rotating and sharing various portfolios during the intervention including managing social media, catering guidelines and cookbook recipe development. As a team, the nutritionists were described as highly resilient and adaptable, working hard together to implement the food literacy program and build health promotion skills of the non-profit organisation.

“I’m proud at how adaptable we were, that we could relate to different communities as well as across the associations channels, from a member all the way up to the state executive.” ^nutritionist (2018)^

The organisation as a whole was not described as adaptable. Descriptions highlighted the organisation as being ridged, inflexible and hierarchical. There was limited description of strategic thinking for the organisation to grow and develop into the future and embrace the program as its core business.

“Then there needs to be more flexibility with managed time and general - I think it’s stupid that you have six, whatever, how many days on the road, and then you come back in and because you’re meant to have a 7.5-hour day, you have to sit in your chair for two hours, even though you can’t function because you’re exhausted. You should just be able to go home.” ^nutritionist (2018)^

### Learning organisation culture

The existing culture in the non-profit organisation influenced the learning of the people who worked (executive management and nutritionists) there. The non-profit organisation was described as not creating a culture that encouraged and supported members to think critically, take risks with new ideas, allowing for mistakes and valuing people’s contributions. The non-profit organisation was described as having limited learning capacity, lacking people with an understanding of health promotion capacity building including skills and processes.

There was evidence that nutritionists tried to shift the organisation towards a “learning organisation” by attempting to improve processes, set up monitoring systems and incorporate health promotion into organisational policy and strategic planning. However, it appeared executive management continued to have a limited understanding about implementing health promotion interventions.

‘They (management) thought that we (nutritionists) were spending their money when they were given money by Queensland Health that directly funds the whole program.’ ^nutritionist (2018)^

The nutritionists were described as having the ability to learn from past experiences and improve their processes over time. The nutritionists reported reflecting and changing the program and developing strategies to build the capacity of their volunteer workforce. Nutritionists themselves also reported learning about capacity and health promotion through professional development workshops and interactions with the evaluation research team. As staff increased their understanding of health promotion practices, they reported applying their learning to how they implemented the community interventions to improve local health outcomes.

“The project team developed the content for the Fabulous Facilitator Training and Networking Weekend based on the feedback that facilitators wanted more support for planning and implementing community activities^”. Newsletter February 2018^

However, they met with resistance from executive management. A lack of organisational culture that promote mutual respect and trust were described. Mechanisms that inhibited organisation learning described by staff included defensive behaviours, protecting current processes and procedures and resistance to updating and refining processes and procedures that would improve the ability of the organisation to implement community interventions.

“I’m not sure that they know exactly what we do, and their support of the program, I feel that it’s still very much us and them; that we’re not completely integrated yet. I’m sure it would only just take time.” ^nutritionist (2018)^

“We’ve planned, we’ve produced, and we’ve delivered, and in the same time frame, they’ve done not much. Why wouldn’t they use our skills and abilities to actually help them do what they need to do?’ ^nutritionist (2018)^

## DISCUSSION

This study explored and described the capacity development of a non-profit organisation to implement a community nutrition intervention over the course of 3-years. Capacity development was found to primarily relate to individual and intrapersonal factors of changing relationships and communication processes. Barriers impeding capacity development included fractious relationships, miscommunication, and resistance to change within the executive management of the organisation. The inability of the organisation to embrace learning was an organisational level factor related to rigid organisational structures and processes. This led to organisational and project management issues, such as inflexible and inefficient procedures and high nutritionist turnover. This highlights the importance of a communication policy being outlined from the start of a health promotion intervention. Despite these barriers the nutritionists did create capacity to support many volunteers to act as health promotion community members. This outcome is attributed to the nutritionists forming strong relationships, communicating, being adaptable and open to change. Having a clear conduit between executive and project staff seems imperative for effective communication and implementation of the program. Relationships appeared to be the bedrock of building capacity for health promotion.

Building relationships and strong communication skills and processes are crucial to capacity (Patton *et al.*, 2000; Thiede, 2005; Anderson-Lewis *et al.*, 2012). Similar to previous research (Ebi and Semenza, 2008; de Groot *et al.*, 2010, Brown *et al.*, 2016), findings from this study indicate relationships and communication were important factors impacting on capacity of the non-profit organisation to implement community nutrition interventions. A pivotal point of change was when capacity development occurred mid-intervention, due to the relationships formed between nutritionists and community volunteers. This is supported by previous research, that found trust and communication, in turn developing engagement and teamwork, was crucial for capacity development and community nutrition intervention dissemination (Anderson *et al.*, 2004; Gilson, 2006; McGlashan *et al.*, 2018). Research also supports that forming connections and relationships takes time (Jagosh *et al.*, 2015). In this study, the types of relationships developed over time and how individuals communicated had a significant influence on workforce development, quality project management and organisational development. Like earlier studies (Economos *et al.*, 2007; de Silva-Sanigorski *et al.*, 2010), it appears that the strong relationships and engagement of community volunteers by nutritionists were key contributors to the positive volunteer workforce development results.

Results from this study indicated limited learning capacity and fair governance impacted on capacity development over time. Previous research suggests organisations should allow individuals to learn from new insights and experiences in order to flexibly and creatively ‘manage’ the expected and the unexpected opportunities. Learning allows for changed understanding based on experiences (Gupta *et al.*, 2010). Executive members from this study did not seem to facilitate effective communication or set an example to enable a learning organisation. Research suggests a more responsive system of governance is required to create a learning organisation that is adaptable and flexible to changing needs (Duit and Galaz, 2008). This includes the establishment of policies, and continuous monitoring of their proper implementation, by the members of the governing body of an organisation. It includes the mechanisms required to balance the powers of the members (with the associated accountability), and their primary duty of enhancing the prosperity and viability of the organisation (Brown *et al.*, 2016). Governance requires responsive processes that show a high degree of transparency and are able to respond to different voices in society (Biermann, 2007), and clear accountability procedures that assign responsibilities to different parties (Connolly and Lukas, 2002). Arguably this non-profit organisation could have been better supported by the funding body, as they had limited experience in the health sector or with implementing a large health promotion intervention.

This research highlights the non-linear, transformative nature of capacity changes over time which is congruent with other research (Greenwood-Lee *et al.*, 2016; Lawrenz *et al.*, 2018; van Herwerden *et al.*, 2019). Aspects such as social, technological, economical, legal, political, and other global factors in which an organisation operate within are changing all the time, so organisations appear to require the ability to adapt when these changes occur. However as recent research suggests, an organisation requires both individuals’ skills, knowledge, and experience and the organisational resources and procedures that enable organisations to use individual expertise productively (DeCorby-Watson *et al.*, 2018; Lawrenz *et al.*, 2018). Findings from this study showed that many people within the non-profit organisation were resistant to change. Therefore, the organisation may not be willing or able to make some changes in its own processes and resources, even if it appears some people are willing to complete individual training, to enhance expertise and increase task performance. This research highlights the interconnection of capacity across individuals in non-profit organisations and that an adaptive capacity framework may be a useful assessment tool, to capture where to focus to improve capacity development of health promotion interventions in non-profit organisations.

This is the first study to apply an adaptive capacity framework to capacity research in the public health or health promotion field. Organisational adaptive capacity has been described as having six dimensions: variety, learning capacity, room for autonomous change, leadership, availability of resources and fair governance (Gupta *et al.*, 2010). Adaptive organisations encourage actors to learn and allow individuals to question roles, rules and procedures that are important for problem solving. This was not found to be the case for the organisation of interest in the present study. Governing has always implied a degree of social learning and of adaptation to changed circumstances (Biermann, 2007). A lack of the ability to adapt, was found to be a limiting factor for capacity development in this study, with limited descriptions of a range of proactive strategies, measures, and instruments to support learning capacity, room for autonomous change and fair governance. Research may exist on the importance of organisational learning and adaptability, but it is rarely assessed. Using an adaptive capacity framework may improve capacity development in health promotion interventions in the future.

Findings further suggest that in future, projects aiming to increase the capacity of an organisation should focus on workforce development across the organisation, particularly with management and decision makers, at the start of the interventions. Further, it is recommended that adaptive capacity of the organisation also be monitored throughout the intervention using an adaptive capacity framework. Training on the need for health promotion adaptive capacity and helping the organisation to focus on embedding adaptive capacity development processes may enhance health promotion interventions to create sustainable outcomes beyond funding periods of interventions. Furthermore, nutritionists employed should have experience in capacity development in community nutrition interventions.

### Strengths and limitations

Our findings were limited to the views of key staff employed by the organisation. The perceptions of funding body or external evaluator outside the organisations were not captured. Document analysis was limited to the newsletters. Other organisational records may have provided additional insights. This study used data from both executive management and project team members and documents. The consistency of findings across the different data sources provides confidence that the researchers inference is reflective of the capacity story.

## FUTURE RESEARCH

Non-profit organisations are key for delivering health promotion interventions but gaining funding to do these types of interventions is very challenging. Assessing organisational capacity during health promotion community interventions by describing adaptive capacity, could help researchers and practitioners identify the resources where they are needed. This may ensure maximum capacity development over the life of a community intervention. Assessing the ability of a non-profit organisation to adapt over time, may provide a more holistic understanding of factors affecting organisational capacity changes over time. This in turn may support funding opportunities of non-profit organisations who can demonstrate they have the capacity to be flexible and the resources to support health promotion community interventions.

## CONCLUSION

Our findings support that capacity is an ever-changing process that is non-linear and transformative. Adaptive capacity is a useful concept to describe and explore organisational capacity changes over time. The process of non-profit organisational capacity development appears to be influenced primarily by individual relationships and communication processes, which in turn impacts on all other capacity determinants. Future community interventions should focus on communication processes and focus on building positive relationships with all members of an organisation, particularly the interactions between executive management of the organisation with nutritionists.

## Supplementary Material

daac074_suppl_Supplementary_Table_S1

daac074_suppl_Supplementary_Table_S2

daac074_suppl_Supplementary_Table_S3
