# Supplementary material for: The role of communication, building relationships, and adaptability in non-profit organisational capacity for health promotion
Source: Health Promot Int. 2022 Jul 28;37(3):daac074. doi: 10.1093/heapro/daac074 (PMC9333191; doi:10.1093/heapro/daac074)
Supplement: daac074_suppl_Supplementary_Table_S1 [file daac074_suppl_supplementary_table_s1.docx]

*Table S1: Overview of non-profit organisation health promotion nutrition program strategies*

| Country Kitchens Program Strategies | Brief Description | Country Kitchens Program  *Roll Out Phases* |
| --- | --- | --- |
| 1. **Facilitator Training for Volunteers** | Initial training of branch member volunteers to become local Country Kitchen volunteers. Content included understanding the health problem of obesity, how to run a Hands-on Nutrition workshop. Follow-up training of local volunteers to support extended learning in health promotion and capacity building occurred. | Phase1 |
| 1. **Hands on Nutrition Workshops (HONWs)** | Nutrition education sessions with a hands-on cooking skills development component for community members. Run by nutritionist and trained volunteer. Sessions were run either three or five times, once a month. |  |
| 1. **Promotional Materials** | A range of recipes, postcards, pens, balloons, stickers, magnetic notepads, tea towels, aprons, Foodie Journals, nutrition education handouts were developed for use in the Hands-on Nutrition workshops, showcases, foodie talks and community activities. |  |
| 1. **Showcases** | Stalls at shows which showcase the Country Kitchens messages and interventions taking place in that community. |  |
| 1. **Foodie Talks** | One-hour session on recipe modification relating specifically to the recipe criteria within the Country Kitchens Healthy Catering Guidelines. |  |
| 1. **Community Interventions** | An activity led by the local volunteer that involved promoting healthy eating or being active. There was no clear expectation that trained volunteers would partner with schools or service organisations. | Phase 2 |
| 1. **Healthy Catering Guidelines** | A booklet designed to promote safe food handling and healthy recipe modification (includes a set of criteria by which to check the healthfulness of a recipe). Includes information for catering for large numbers. |  |
| 1. **Healthy Cookbooks** | Cookbooks released which contain recipes that meet the criteria within the Healthy Catering Guidelines. Recipes contributed by local members. |  |
| 1. **Capacity Workshop for volunteers** | Capacity training run by nutritionists for trained volunteers who were tasked with implementing community interventions. Occurred in the last year of the three-year intervention. |  |

*Adapted from (Palermo, van Herwerden et al. 2018)*
