# Supplementary material for: The role of communication, building relationships, and adaptability in non-profit organisational capacity for health promotion
Source: Health Promot Int. 2022 Jul 28;37(3):daac074. doi: 10.1093/heapro/daac074 (PMC9333191; doi:10.1093/heapro/daac074)
Supplement: daac074_suppl_Supplementary_Table_S2 [file daac074_suppl_supplementary_table_s2.docx]

*Table S2: Semi-structured interview questions 2016 and 2018, capacity prompts, question* *logic*

| Interview Question | Capacity prompts and logic^#^ |
| --- | --- |
| 1. In your words, how would you describe the program? | Leadership potential of executive |
| 1. Tell me about your experience of program? | Organisational development occurred (support from division, branches, governance structures, membership engagement) Resources, Quality, satisfaction. |
| 1. In your opinion, how effective has program? What, or how has the program, been effective? | Program impacts, Partnerships  Organisational development. What do they describe as effective: Running food literacy programs or organisational change? |
| 1. Can you describe any barriers or difficulties you encountered with the program? | Organisational, community, individual barriers described? reflect on social-ecological model |
| 1. Thinking back to when the program first commenced, has your attitudes or expectations of the program changed in any way? Can you describe? | Reflective practice, learnings, descriptions of capacity changes (individual, community, organisational) |
| 1. What role or impact do you believe the program has had on the non-profit organisation? | Capacity for organisation to host  Confidence capacity can be built  Workforce development, Resources |
| 1. When thinking about the program, can you think of a maximum of 5 people that come to mind? * | Networks/cooperating/coordinating/collaborating (Partnerships), Workforce development, thinking strategically- big picture or small picture? who do they name- managers, nutritionists, volunteers? Leaders? |
| 1. Do you think the program enhancing the capacity of the organisation, and if so, in what ways? * | Knowledge, intelligence, reflective practice policies and procedures or systems thinking capacity |
| 1. Do you have any other comments? | Opportunity to cover points important to interviewee not covered by interview questions |

#(MacLellan-Wright, Anderson et al. 2007, Kostadinov, Daniel et al. 2015, van Herwerden, Palermo et al. 2019) (Gupta, Termeer et al. 2010)

*2018 questions only
