# Supplementary material for: The role of communication, building relationships, and adaptability in non-profit organisational capacity for health promotion
Source: Health Promot Int. 2022 Jul 28;37(3):daac074. doi: 10.1093/heapro/daac074 (PMC9333191; doi:10.1093/heapro/daac074)
Supplement: daac074_suppl_Supplementary_Table_S3 [file daac074_suppl_supplementary_table_s3.docx]

*Table S3: Capacity coding framework*

| **Deductive capacity categories** | **Description(s) codes** |
| --- | --- |
| Community development ^2-5^ | Community readiness to mobilise  Community structures  Equity (acknowledging health inequalities and prioritising activities with those who stand to benefit the most/who’s needs are greatest)  Participation  Role of external supports  Sense of community |
| Fair Governance ^9^ | Legitimacy  Equity  Responsiveness  Accountability |
| Intelligence^2,8^ | Asking why?  Addressing the root causes of the issue(s) targeted by the project  Involving the target population in the process of asking why? |
| Leadership^1, 9^ | Communication skills  Creative collaboration  Entrepreneurial  Personal growth and development  Political and social change strategies  Systems and strategic thinking  Team learning  Visioning for the future and reformist leaders |
| Learning Capacity^9^ | Trust  Single loop learning  Double loop learning  Discuss doubts  Organisational memory |
| Organisational Development^1^ | Informal organisational culture  Information systems, monitoring and evaluation  Management support and commitment  Organisational management structure  Policies and strategic planning  Quality improvement systems  Recognition and reward systems |
| Partnerships^1,6,7^ | Evaluation  Planning and implementing  Relationships  Shared goals  Sustained outcomes |
| Resources^1, 9^ | Decision making resources  Environmental* resources  Financial resources  Human resources  Information resources |
| Room for autonomous change^9^ | Continuous access to information  Act according to plan  Capacity to improvise |
| Quality Project Management^3,7^ | Careful planning, monitoring, evaluation and adjustment as required (i.e. adaptability)  Developing an organisational wide culture of quality  Encouraging and rewarding, rather than discouraging, new ideas  Providing supervision and support for staff and volunteers  Team work and empowerment for all in the organisation |
| Variety^9^ | Variety of problem frames  Multi-actor, multi-level, multi-sector  Diversity of solutions  Redundancy (duplication) |
| Workforce Development^1^ | On the job training (formal and informal)  Professional development opportunities  Performance management systems |

1(.Liberato et al., 2011), 2. (Health, 2001), 3. (MacLellan-Wright et al., 2007), 4. (University of Kansas, 2018) , 5. (Kostadinov et al., 2015), 6.(VicHealth, 2011) 7. (Mann, Gordon, & MacLeod, 2009) 8.(Hughes & Margetts, 2012) 9. (Gupta et al., 2010)
